# Multiple Giant Splenic Artery Aneurysms With Chronic Portal and Splenic Vein Thrombosis: A Case Report of a Rare Surgical Challenge

**DOI:** 10.7759/cureus.93006

**Published:** 2025-09-23

**Authors:** Suhas Devanathan, Nitin AR Rao, Avinash Balekuduru

**Affiliations:** 1 Surgery, M. S. Ramaiah Medical College, Bangalore, IND; 2 Surgical Gastroenterology, M. S. Ramaiah Medical College, Bangalore, IND; 3 Medical Gastroenterology, M. S. Ramaiah Medical College, Bangalore, IND

**Keywords:** distal pancreatectomy, open splenectomy, portal vein thrombosis, splanchnic thrombosis, splenic artery aneurysm, splenomegaly, vascular surgery, visceral artery aneurysm

## Abstract

Splenic artery aneurysms (SAAs) are common visceral artery aneurysms with low prevalence. They are typically asymptomatic and discovered on incidental grounds. Multiple SAA, particularly large or giant ones, are extremely rare and are highly dangerous due to their propensity to rupture, particularly in the context of portal hypertension and thrombosis. We report a challenging and unusual case of a 36-year-old female who presented with epigastric pain and was found to have splenomegaly with several large SAAs and chronic splenic and portal vein thrombosis. She was successfully treated with open splenectomy, splenic artery ligation, and distal pancreatectomy. This case highlights the difficulty in diagnosis, surgical complexity, and rarity of multiple SAAs under chronic thrombosis, as well as the necessity of early management to prevent mortal consequences.

## Introduction

Splenic artery aneurysms (SAAs) are the most frequently encountered aneurysms of visceral arteries, accounting for approximately 60% of the total splanchnic artery aneurysms. Despite this, they are sporadic, with a prevalence rate of 0.01% to 10.4% in autopsy and around 0.78% in angiography reports [1]. SAAs may be asymptomatic and incidentally detected during imaging for other abdominal complaints. However, their clinical relevance lies in the potentially catastrophic complications of rupture, which carry a mortality rate of as much as 25% and an even higher one in pregnancy, with fetal mortality rates of nearly 95% [2].

The majority of SAAs occur in isolation and are small, often less than 2 cm in diameter. Giant aneurysms (>5 cm) and multiple SAAs are sporadic. The prevalence of numerous SAAs has been reported from 0.02% to 0.1%. Risk factors for aneurysm development are portal hypertension, pregnancy, cirrhosis, systemic hypertension, liver transplantation, and vascular/connective tissue diseases. Their association with portal hypertension and splenic vein thrombosis, the underlying conditions that lead to collateral development and increased aneurysm rupture risk owing to the hemodynamic changes, is a significant concern [3].

This case is significant in that it represents the coexistence of multiple large SAAs with chronic thrombosis of the splenic and portal veins, an extremely uncommon condition. The case challenged diagnosis and surgery due to anatomical site, multiplicity, size, and associated vascular thrombosis.

## Case presentation

A 36-year-old female presented to the surgical gastroenterology outpatient department of our tertiary care facility with a one-month history of left upper quadrant abdominal pain in May 2025. It was sudden in onset, pricking in character, intermittent, and moderate in severity. The pain was located in the left hypochondrium with no radiation. There were no relieving or aggravating factors. She provided no history of vomiting, fever, distension of the abdomen, jaundice, alteration in bowel habits, or loss of weight. She had no history of trauma, comorbid illnesses such as hypertension, diabetes, or chronic liver disease, or drug allergies. She had neither received any previous surgery nor blood transfusions.

During physical examination, the patient was conscious, alert, and oriented to time, place, and person. She was well-nourished and moderately well-built. Her vitals were within normal range, with a heart rate of 80 beats per minute, blood pressure of 134/80 mmHg, and a respiratory rate of 18 breaths per minute. Fever, pallor, icterus, cyanosis, clubbing, or lymphadenopathy were not present.

An abdominal examination revealed that the abdomen was soft and not distended. The umbilicus was central, and no scars, dilated veins, or sinuses were evident. Palpation revealed left hypochondrial tenderness with palpable splenomegaly. Guarding and rigidity were not observed. The percussion was tympanic, and bowel sounds were auscultated. Other systems, like the cardiovascular, respiratory, and central nervous systems, were otherwise unremarkable.

**Figure 1:**
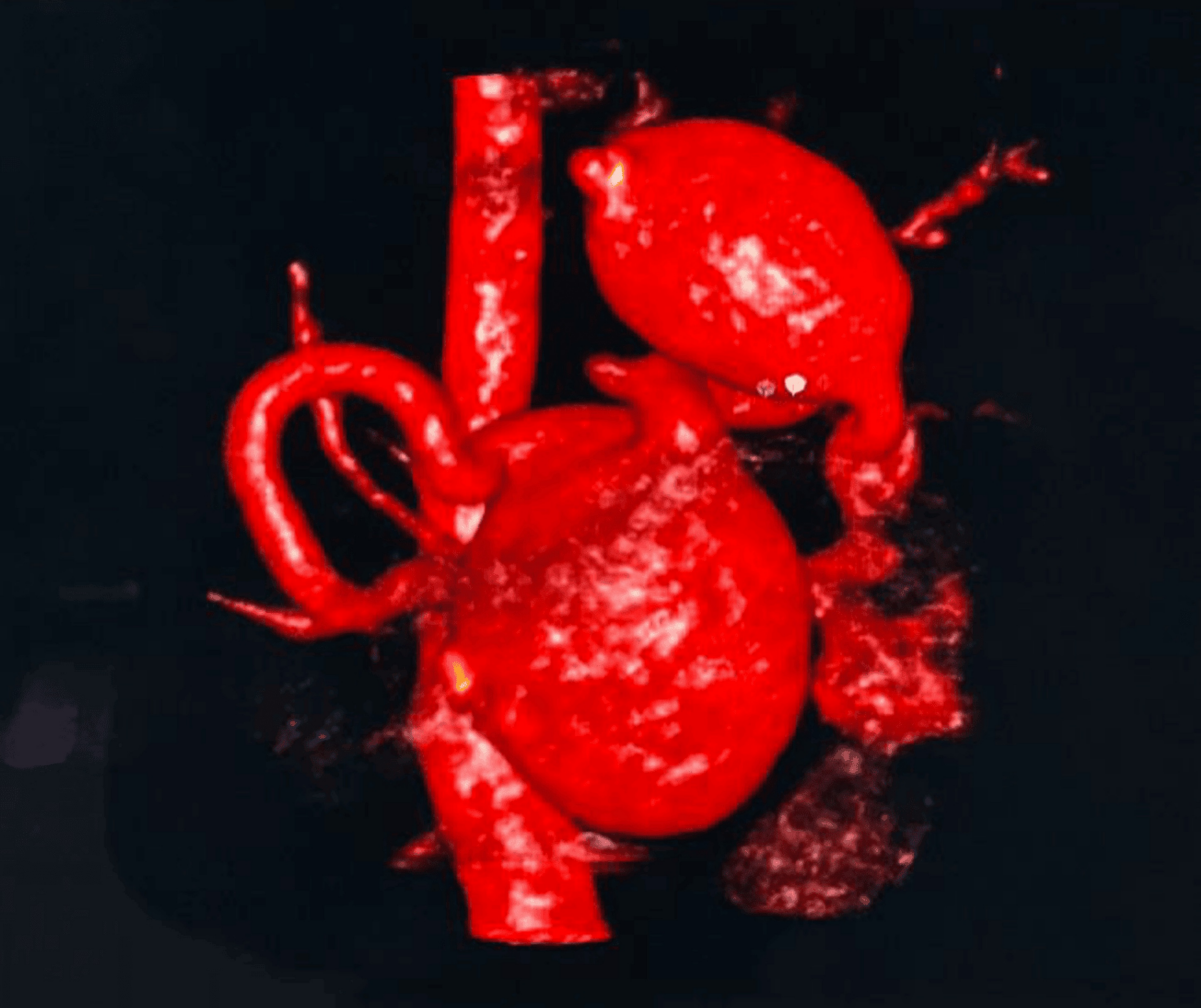
Three-dimensional reconstruction image showing multiple splenic aneurysms

The initial laboratory investigations revealed normal liver and renal function tests, a mild decrease in hemoglobin, and a normal coagulation profile. Considering the local tenderness and palpable splenomegaly, a contrast-enhanced computerized tomography scan with 3D reconstruction (Figure 1) was done, which revealed marked splenomegaly along with three SAAs. Chronic thrombosis in the portal and splenic veins was noted, and a large magnitude of collateral circulation was observed.

A multidisciplinary team, including surgical gastroenterology, anaesthesia, and vascular surgery, evaluated the patient. The patient underwent optimisation for elective open splenectomy with ligation of the splenic artery. Vaccinations against encapsulated bacteria (pneumococcal, meningococcal, and *Haemophilus influenzae* type B) were administered.

Informed written consent was obtained after a detailed discussion of the disease's nature, surgical procedure, potential complications, and need for postoperative ICU stay and blood transfusion. The patient was taken up for surgery under general anaesthesia.

Surgery began with a left subcostal incision. Splenomegaly was encountered during intraoperative exploration. Three large SAAs were encountered. The most proximal aneurysm was approximately 4×3 cm. Two additional aneurysms, located near the origin of the splenic artery, measured 7×3 cm and 5×6 cm (bilobed) and were adherent to the pancreas. There were multiple splenic artery collaterals present in the diaphragm, as well as dilated short gastric arteries. The liver was grossly normal. No peritoneal metastases or other abnormalities were identified.

**Figure 2:**
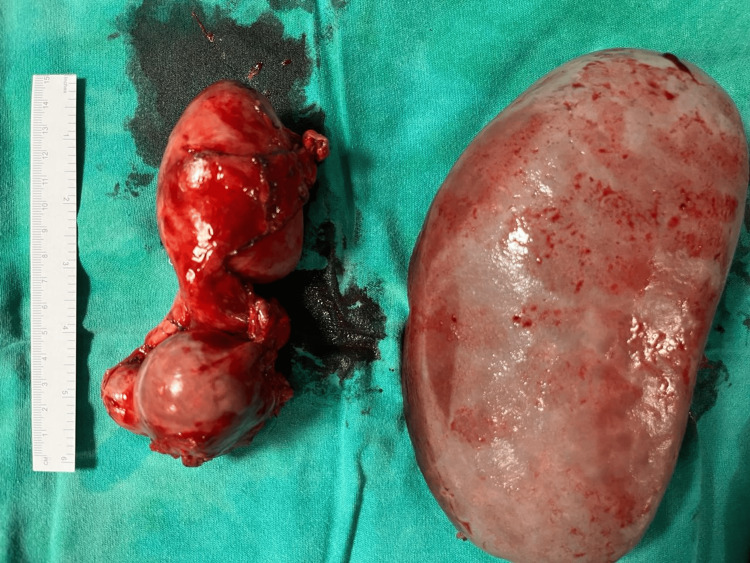
Specimen images Spleen and distal pancreas seen in figure.

Open splenectomy and splenic artery ligation were performed. Distal pancreatectomy was performed to ensure secure resection of the aneurysm and achieve hemostasis. Specimen images are seen in Figure 2. Hemostasis was judicious, bearing in mind the vascularity and collaterals in the region. Intraoperatively, the patient received two units of single-donor platelets, one unit of random-donor platelets, and one unit of packed red blood cells (PRBC). A drain was placed, and the abdomen was closed in layers.

She was shifted to SICU for close observation in the early postoperative phase. She remained hemodynamically stable and managed well with supportive measures. Postoperatively, she received four units of fresh frozen plasma and a pint of PRBC with consideration of low hemoglobin. Drain output was serosanguinous in character and decreased gradually. The removal of the abdominal drain was done on postoperative day 7.

Histopathological examination of the resected spleen showed chronic venous congestion. The aneurysms were associated with focal non-occlusive mural thrombus in the splenic artery, which was consistent with the radiologic finding of chronic vascular thrombosis. Surgical staples were removed after proper healing of the wound.

The patient had mild left hypochondriac pain during the postoperative period. Follow-up ultrasonography of the abdomen and pelvis revealed ongoing chronic portal vein thrombosis and thrombosed splenic collaterals. A vascular surgical opinion was sought, and the patient was started on subcutaneous enoxaparin 0.6 mg, later converted to oral antiplatelet with aspirin 75 mg daily. Immunizations (pentavalent, Menveo, and Prevenar-13) were administered according to the post-splenectomy protocol.

She was discharged in a stable condition with appropriate oral medications and follow-up instructions in surgical gastroenterology, vascular surgery, and hematology outpatient clinics. She was advised to continue oral antiplatelet therapy, iron and multivitamin supplements, and nutritional protein supplementation. She was educated regarding post-splenectomy infection risks and red-flag signs requiring emergency consultation. In the post-discharge follow-up, the patient remained stable and was doing well. She continued her prescribed medications, including oral antiplatelet therapy, and was monitored for any signs of complications.

## Discussion

SAAs, though the most common of visceral artery aneurysms, remain relatively rare, with an estimated prevalence of 0.1% in the general population. They are often discovered incidentally, as they are typically asymptomatic. When symptoms do occur, they tend to be nonspecific, such as upper abdominal pain, nausea, or left upper quadrant pain, as was observed in this patient. These findings align with the broader literature, which commonly reports that SAAs are typically asymptomatic and are often found during imaging for other conditions [4].

Interestingly, although the literature emphasizes the incidental nature of these aneurysms, there are instances where patients, like in this case, present with vague abdominal complaints. The mild left hypochondriac pain observed postoperatively could be an expression of the patient’s underlying vascular condition, though it did not escalate into a major complication. This emphasizes the need for heightened clinical suspicion and timely imaging to detect such vascular anomalies before they evolve into more dangerous conditions, as some cases can go unnoticed until rupture occurs.

The most feared complication of an SAA is rupture, which can present as acute abdominal pain, hypotension, or hemorrhagic shock. Ruptured SAAs have a reported mortality rate ranging from 10% to 25%, with an even more severe prognosis in pregnant women, where the mortality rate may rise as high as 70%. This mirrors the findings in several studies that underscore the high mortality associated with rupture. The risk factors for rupture are well-established in the literature and include aneurysm size (>2 cm), rapid growth, pregnancy, portal hypertension, liver transplantation, and symptomatic presentation [5]. The patient in this case, however, did not experience rupture, likely due to timely intervention and appropriate monitoring. The fact that this patient was not pregnant or suffering from additional risk factors such as portal hypertension could have also contributed to the favorable outcome.

Regarding treatment, the literature generally supports endovascular therapy, such as coil embolization or stent-grafting, as the preferred treatment for isolated aneurysms. However, in cases like this, where multiple aneurysms are involved and there is adjacent organ involvement (e.g., pancreas), open surgical resection is often necessary. This case supports the concept that, despite advancements in endovascular techniques, complex or multi-aneurysmal presentations, particularly those with involvement of nearby structures like the pancreas, still warrant open surgery. The decision to perform a distal pancreatectomy in this case aligns with current guidelines, which indicate that resection is often the best approach when there is a significant risk of aneurysm rupture or complications in areas such as the pancreatic tail or body. This contrasts with the more common, less invasive treatment strategies for isolated SAAs, suggesting that a tailored, case-by-case approach is essential in managing complex cases [6].

Furthermore, the importance of prophylactic antibiotics and post-splenectomy vaccination is well-documented in the literature to prevent Overwhelming Post-splenectomy Infection (OPSI), a serious and potentially life-threatening condition. This patient's post-splenectomy immunization protocol, which included vaccines such as Pentavalent, Menveo, and Prevenar-13, reflects standard practice in post-splenectomy care. Although OPSI is rare, its devastating potential makes it essential for all patients who undergo splenectomy to be educated about the risk and follow a structured vaccination regimen [7].

This case underlines the importance of elevated clinical suspicion, careful imaging, and prompt multidisciplinary intervention when dealing with rare vascular malformations like SAAs. The findings are consistent with existing scientific literature, particularly regarding the treatment approach for complex presentations with adjacent organ involvement, where open surgery remains the gold standard. Moreover, the overall excellent surgical outcome in this patient highlights the continued efficacy of open surgical intervention in complex vascular cases, especially when endovascular solutions are not viable due to the aneurysm’s location or size. This stands in contrast to isolated cases where minimally invasive methods may be sufficient, reinforcing the need for personalized treatment based on the patient's specific condition.

## Conclusions

This case represents a novel and unusual occurrence of multiple giant SAAs in conjunction with chronic portal and splenic vein thrombosis. Accurate diagnosis and appropriate surgical therapy were critical to prevent potentially fatal complications. This case highlights the value of clinician suspicion for such rare vascular abnormalities, especially in young females presenting with nonspecific abdominal discomfort. Multidisciplinary care, individualized surgical planning, and vigilant postoperative management are all of paramount significance in achieving satisfactory results under such high-risk conditions.
